# Comparison of iron chelation effects of deferoxamine, deferasirox, and combination of deferoxamine and deferiprone on liver and cardiac T2* MRI in thalassemia maior

**DOI:** 10.22088/cjim.8.3.159

**Published:** 2017

**Authors:** Shahla Ansari, Azita Azarkeivan, Ghasem Miri-Aliabad, Saeed Yousefian, Tahereh Rostami

**Affiliations:** 1 Department of Hematology-Oncology, Ali Asghar Children’s Hospital, Iran University of Medical Sciences, Tehran, Iran.; 2 Transfusion Research Center, High Institute for Research and Education in Transfusion Medicine, Department of Thalassemia Clinic, Tehran, Iran.; 3 Children and Adolescent Health Research Center, Zahedan University of Medical Sciences, Zahedan, Iran.; 4 Department of Hematology-Oncology, Isfahan University of Medical Sciences, Isfahan, Iran.; 5 Department of Hematology-Oncology, Tehran University of Medical Sciences, Tehran, Iran.

**Keywords:** Thalassemia major, Deferoxamine, Deferasirox, Deferiprone

## Abstract

**Background:**

Cardiac complications due to iron overload are the most common cause of death in patients with thalassemia major. The aim of this study was to compare iron chelation effects of deferoxamine, deferasirox, and combination of deferoxamine and deferiprone on cardiac and liver iron load measured by T2* MRI.

**Methods:**

In this study, 108 patients with thalassemia major aged over 10 years who had iron overload in cardiac T2* MRI were studied in terms of iron chelators efficacy on the reduction of myocardial siderosis. The first group received deferoxamine, the second group only deferasirox, and the third group, a combination of deferoxamine and deferiprone. Myocardial iron was measured at baseline and 12 months later through T2* MRI technique.

**Results:**

The three groups were similar in terms of age, gender, ferritin level, and mean myocardial T2* at baseline. In the deferoxamine group, myocardial T2* was increased from 12.0±4.1 ms at baseline to 13.5±8.4 ms at 12 months (p=0.10). Significant improvement was observed in myocardial T2* of the deferasirox group (p<0.001). In the combined treatment group, myocardial T2* was significantly increased (p<0.001). These differences among the three groups were not significant at the 12 months. A significant improvement was observed in liver T2* at 12 months compared to baseline in the deferasirox and the combination group.

**Conclusion:**

In comparison to deferoxamine monotherapy, combination therapy and deferasirox monotherapy have a significant impact on reducing iron overload and improvement of myocardial and liver T2* MRI.

Beta-thalassemia major is a hereditary disorder of hemoglobin which results in impaired beta-globin chain synthesis and leads to hemolytic anemia (1). Regular blood transfusions are necessary for long-term survival of patients, but iron deposition gradually occurs in various tissues of the body (2, 3). Since no mechanism exists for active excretion of excess iron of the body, iron overload leads to its deposition in major organs including liver, spleen, myocardium, and endocrine system and thus may cause complications such as growth retardation, liver disease, diabetes mellitus, cardiomyopathy, and so on (4). Heart disease, resulting from iron deposition in the myocardium, is the most common cause of death in these patients (5).

Iron chelating agents including deferoxamine, deferiprone, and deferasirox reduce iron overload in these patients in different degrees and therefore reduce morbidity and mortality, including cardiac complications (3, 6). Serum ferritin can be used to estimate the amount of iron storage, but it can be influenced by various factors such as fever, infection, inflammation, hemolysis, liver disease and acid ascorbic deficiency (7, 8). The most reliable method for assessment of body iron storage is to examine iron in liver biopsy sample. However, due to its invasive nature, this procedure is not useful for routine follow-up of patients and also it does not give information on the amount of iron deposited in the myocardium (7). The T2* MRI is a non-invasive method for quantifying cardiac iron overload and for following the response to iron chelators (9, 10). Several studies were conducted about the effectiveness of iron chelators of deferoxamine and deferiprone combination therapy and deferasirox monotherapy which had different results (8, 9). The aim of this study was to compare the effect of iron removal of deferoxamine, deferasirox, and combination of deferoxamine and deferiprone on cardiac and liver iron load, measured by T2* MRI in patients with thalassemia major.

## Methods

**Figure 1:**
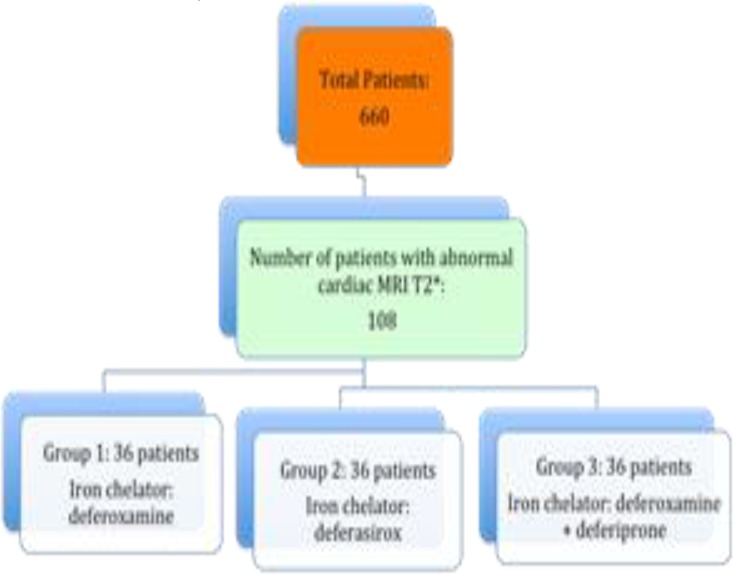
Flowchart of study and patients selection

This interventional and semi-experimental study was carried out at Ali Asghar Children’s Hospital and Zafar Adult Thalassemia Clinic, Tehran, on transfusion-dependent major thalassemic patients with over 10 years of age (10-33) in 2012-2013 (IRCT No: IRCT 2014092219255N1). Among patients with abnormal cardiac T2* MRI (<20 ms) and without obvious symptoms of heart failure, 108 patients (three groups of 36 each) similar in terms of age, gender, heart siderosis, and mean ferritin levels were selected (fig 1). 

This study was approved by the Ethics Committee of Tehran University of Medical Sciences, Tehran, Iran (no: 91/d/130/385). A written consent was obtained from patients or their parents for their participation in the study. The first group was treated with subcutaneous infusion of 30-50 mg/kg/day deferoxamine during 8-12 hrs for at least 5-6 nights a week using a pump. The second group received 20-40 mg/kg/day deferasirox as a single oral dose before the first meal. The third group was treated with subcutaneous infusion of 30-50 mg/kg/day deferoxamine during 8-12 hrs for at least 5-6 nights a week using a pump and 75-100 mg/kg/day oral deferiprone in three divided doses. The iron chelation regimen continued for at least one year in all groups. Patients receiving deferasirox were examined for liver and kidney functions before beginning the treatment and then once a month throughout the treatment. Those receiving deferiprone were tested for their blood cell count at the start of treatment and then once a week at the beginning the treatment course. 

All patients were routinely examined every 6 months in terms of serum ferritin level, liver, kidney, and endocrine function, and virologic studies. Heart and liver MRI T2* was performed at the end of the first year of treatment. Those patients who discontinued the treatment earlier than 12 months and/or used another chelator were excluded from the study. Patients with severe liver disease, kidney disease, history of neutropenia, history of previous adverse effects of any chelator, inability to perform T2* MRI due to pacemaker, pregnancy, and fear of closed environment were also excluded. The patients were selected from 660 patients (over 10 years old) who had the inclusion criteria. 

The sample selection was based on the convenient sampling method. Each patient who fulfilled the inclusion criteria and agreed to be included participated in this study. MRI 1.5 T scanner was used for imaging (breath-hold multiecho) whose sequence and time was 5-10 days after transfusion. Cardiac T2* MRI values ​​were considered as follows: normal (>20 ms), mild (14-20 ms), moderate (10-14 ms), severe (<10 ms). Average increase of T2* MRI values compared to baseline ​​was considered as an improvement, and its reduction was considered as increment in heart iron overload. Serum ferritin was measured through RIA in the absence of viral or bacterial infection within 2 weeks prior to the test. 

The primary objective of this study was to change the myocardial T2* from baseline to the month 12 in each of the treatment groups and the next objective was to change the liver T2* and serum ferritin compared to the baseline. Statistical analyses were performed using SPSS Version 16 software. 

Quantitative variables were shown as mean±SD. To compare the mean values ​​between the three groups, one way ANOVA test was used. To compare the mean values ​​in each group at baseline and at 12 months, paired *t*-test was used. To compare the qualitative and classified variables, chi-square test was used. A p<0.05 was considered of statistical significance.

## Results

Patient baseline characteristics are shown in table 1. All three groups were well matched in terms of serum ferritin and heart and liver iron load at baseline. For other parameters, they were also well matched except for total bilirubin. The mean iron chelator dose was 34.4±7.4 mg/kg/day in deferoxamine group, 29.8±6.5 mg/kg/day in deferasirox group, and 36.5±8.8 mg/kg/day and 76.8±6.2 mg/kg/day for deferoxamine and deferiprone in the combination group, respectively. Four patients, including 2 from deferoxamine group, one from deferasirox group, and one from combination group left the study before 12 months. The most common reported adverse events were gastrointestinal symptoms including nausea, vomiting, and abdominal pain that occurred in deferoxamine, deferasirox, and combination therapy groups, in 17.6%, 34.4%, and 22.9% of the patients, respectively. These side effects were mild and transient. In deferoxamine group 5 (14.7%) patients had local skin reactions at the injection site. Other side effects were rarely reported.

**Table 1:** Demographic, clinical and laboratory findings of patients with thalassemia major in three treatment groups

**Parameter**	**Deferoxamine group (n=34)**	**Deferasirox group(n=35)**	**Combined group** * **(n=35)**	**P-value**
Mean age±SD, years	25.3 ± 5.1	22.2 ± 7.6	24.6 ± 5.2	0.09
Males, no. (%)	18 (52.9)	15(42.9)	16(45.7)	0.68
Splenectomy, no. (%)	20(58.8)	15(42.9)	22(62.9)	0.20
Hepatitis C positive (%)	9(26.5)	9(25.7)	14(40)	0.34
Myocardial T2*, ms	12.0±4.1	13.0±4.5	11.6±3.8	0.38
Liver T2*,ms	5.5±6.6	3.4±3.1	4.8±5.1	0.23
Serum ferritin, ng/ml	2794±2310	3112±2549	2817±2134	0.81
Hemoglobin, g/dl	9.4±1.3	9.4±1.1	9.6±1.3	0.78
ANC×10^9^/L	6.3±2.5	5.2±2.6	5.0±2.5	0.09
Platelets, ×10^9^/L	510±247	473±263	463±258	0.73
ALT, IU/L	41±16	36±17	42±22	0.43
AST, IU/L	38±23	41±30	51±30	0.15
Bilirubin total, mg/dl	2.5±1.3	2.1±1.0	1.9±0.8	0.03
Creatinine, mg/dl	0.64±0.19	0.69±0.17	0.67±0.11	0.39

* Deferoxamine + deferiprone

Table 2 shows the variations of mean ferritin and liver and cardiac T2* MRI 12 months after treatment in comparison to the baseline. In the deferoxamine group, no significant change was seen in the mean serum ferritin (p=0.32) and liver (p=0.72) and cardiac T2* MRI (p=0.10) 12 months after treatment compared to baseline. Changes in the mentioned parameters were significant in the deferasirox group. In the combination group, there was a significant improvement in cardiac (p=0.001) and liver T2* MRI (p=0.005) relative to the baseline and the mean serum ferritin decreased compared to baseline, although these changes were not significant. Table 3 shows the mean serum ferritin and liver and cardiac T2* MRI in the three groups at baseline and 12 months later. No significant differences existed between the three groups in terms of cardiac and liver T2* MRI and the mean serum ferritin. No significant change occurred in serum creatinine and alanine aminotransferase (ALT) in any of the groups in month 12 compared to baseline except for hemoglobin in the deferoxamine group (table 4). In addition, no significant change occurred in other parameters such as white blood cell count (WBC), platelet count, and aspartate aminotransferase (AST) in any of the groups at month 12 compared to baseline. No significant difference was seen in the absolute neutrophil count (ANC) in the deferasirox and combination therapy group in month 12 compared to baseline. Nonetheless, in the deferoxamine group this difference was significant (6.3±4.3×10^9^ /L at baseline versus 5.3±2.5×10^9^ /L at 12 months, P=0.004). Random urine protein to creatinine ratio in patients treated with deferasirox was 0.10±0.04 and 0.11±0.05 before and after a 12- month treatment, respectively (P=0.19). This difference was not statistically significant. There was no significant difference in hemoglobin, ANC, platelet count, creatinine, AST, ALT, and bilirubin between groups at 12 months.

**Table 2:** Changes in cardiac and liver T2* MRI and serum ferritin at baseline and at 12 months in each group

Liver MRI T2* (ms)		Cardiac MRI T2* (ms)		**Ferritin (ng/ml)**		**Variable** **Group**	
**12 month**	**Baseline**	**12 month**	**Baseline **	**12 month**	**Baseline**		
6.2±9.5	5.5±6.6	13.5±8.4	12.0±4.1	2974±2894	2794±2310	Mean±SD	Deferoxamine
0.72		0.10		0.32		P- value	
4.9±5.3	3.4±3.1	17.5±7.1	13.0±4.5	2488±2088	3112±2549	Mean±SD	Deferasirox
0.01		<0.001		0.005		P- value	
7.7±9.5	4.8±5.1	16.8±9.9	11.6±3.8	2681±2281	2817±2134	Mean±SD	Deferoxamine +Deferiprone
0.005		0.001		0.29		P- value	

**Table 3:** Differences in the mean ferritin, liver and cardiac T2* MRI at baseline and at 12 months between groups

**P- value**	**Deferoxamine** **+Deferiprone**	**Deferasirox**	**Deferoxamine**	**Group** **Variable**	
0.38	11.6±3.8	13.0±4.5	12.0±4.1	Baseline	Cardiac MRI T2* (ms)
0.09	16.8±9.9	17.5±7.1	13.5±8.4	At 12 month	
0.23	4.8±5.1	3.4±3.1	5.5±6.6	Baseline	Liver MRI T2*(ms)
0.39	7.7±9.5	4.9±5.3	6.2±9.5	At 12 month	
0.81	2817±2134	3112±2549	2794±2310	Baseline	Ferritin (ng/ml)
0.70	2712±2425	2488±2088	2974±2894	At 12 month	

## Discussion

Cardiomyopathy and heart failure due to iron deposition in myocardium as a result of repeated and prolonged blood transfusion and increased iron absorption from the gastrointestinal tract is the leading cause of death in patients with thalassemia major (1). Surveillance and monitoring of patients in terms of iron overload and therefore an appropriate and timely treatment with iron chelators is extremely important in prevention of these complications and reducing patients’ mortality and morbidity. Several methods exist for monitoring patients for iron overload including serum ferritin measurement, liver and myocardial T2* MRI, and liver biopsy, each having advantages and disadvantages. Myocardial and liver MRI T2* is a reliable and non-invasive method that can be used for this purpose but it is not available everywhere (11-13). Deferoxamine is a chelator used from 1970 as the standard iron chelator in these patients but despite the improvement in their survival, a high proportion of them still suffer from cardiac and liver iron overload thus resulting in mortality and morbidity (12, 14). Several studies were performed on the effects of other iron chelators such as deferiprone and deferasirox, as well as combination therapy which had different results (14). 

In the present study, an improvement was seen in myocardial and liver T2* MRI in deferoxamine group compared to baseline, but it was not significant. Serum ferritin level also increased compared to baseline. The study of Anderson LJ et al. on thalassemia major patients showed that the mean myocardial iron was lower and ejection fraction was higher in deferiprone group than deferoxamine group (15). In patients treated with deferasirox, a significant improvement was observed in cardiac and liver MRI T2* and in the mean serum ferritin from the beginning of treatment to 12 months later. These findings indicate that deferasirox is effective in improving myocardial and hepatic siderosis. Consistent with our research, in the study of Pathare A et al., a significant reduction in serum ferritin, as well as a remarkable improvement in cardiac T2* MRI were seen in thalassemia major patients with severe iron overload treated with deferasirox (9). 

In two prospective studies, the effectiveness of deferasirox was shown in the prevention and removal of myocardial iron (16, 17). Another study demonstrated the failure of deferasirox in myocardial iron removal in patients with severe hepatic iron overload, while being effective in mild to moderate cases (18). Although, Gao HY showed that deferasirox is effective in reducing serum ferritin and liver iron, while it is ineffective in removal of myocardial iron (19). In the combined treatment group, a significant improvement was also observed in cardiac and liver MRI T2* from baseline to 12 months later, but a significant reduction in mean serum ferritin was not observed.

The study of Daar S *et al*. also showed that combination therapy with deferoxamine and deferiprone in patients with thalassemia major with iron overload significantly decreased ferritin level and improved ejection fraction (8). In a study conducted by Origa R *et al*., the efficacy of combination therapy with deferiprone and deferoxamine was compared to deferoxamine monotherapy. In that study, it was found that combination therapy significantly reduced serum ferritin and negative iron balance and improved heart function (20). Tanner MA *et al*. observed a significant improvement in myocardial and liver MRI T2* and left ventricular ejection fraction in patients receiving combination therapy with deferoxamine and deferiprone (21). 

In two similar studies, a significant improvement in cardiac T2* MRI and serum ferritin was observed compared to deferoxamine monotherapy (22, 23). Thus, our results like the mentioned studies show that adding deferiprone to deferoxamine has favorable effects on the improvement and reduction of myocardial and hepatic siderosis. Combination therapy is safe and well tolerated and should be considered in those patients that deferoxamine alone is not effective in controlling iron overload. Since a number of patients have poor compliance to the use of parenteral chelators or combination therapy, and also the effectiveness of deferasirox in reducing the iron load of the whole body, heart, and liver has been shown in several studies, like the present study, this medicine could be an appropriate alternative to prevention and treatment of total body iron load, heart and liver siderosis. Yet, monitoring its continuing effectiveness requires controlled and long-term studies with more patients. In conclusion, Overall, it can be concluded that combination therapy with deferoxamine and deferiprone and deferasirox monotherapy has significant effect on reducing iron load and improvement of myocardial and liver T2* MRI compared to deferoxamine monotherapy.
